# Recombinant Immunotoxin 4D5scFv-PE40 for Targeted Therapy of HER2-Positive Tumors

**Published:** 2015

**Authors:** E. A. Sokolova, O. A. Stremovskiy, T. A. Zdobnova, I. V. Balalaeva, S. M. Deyev

**Affiliations:** Lobachevsky State University of Nizhny Novgorod, pr. Gagarina 23, 603950, Nizhny Novgorod, Russia; Shemyakin–Ovchinnikov Institute of Bioorganic Chemistry, Russian Academy of Sciences, ul. Miklukho-Maklaya 16/10, 117997, Moscow, Russia

**Keywords:** recombinant immunotoxin, 4D5scFv, Pseudomonas exotoxin A, HER2 tumor marker, targeted therapy

## Abstract

Recombinant immunotoxins are extremely promising agents for the targeted therapy of tumors with a certain molecular profile. In this work, we studied the properties of a new recombinant HER2-specific immunotoxin composed of the scFv antibody and a fragment of *Pseudomonas *exotoxin A (4D5scFv-PE40). High affinity of the immunotoxin for the HER2 tumor marker, its selective cytotoxicity against HER2-overexpressing cells, and its storage stability were demonstrated. The 50% inhibitory concentration (IC_50_) of the 4D5scFv-PE40 immunotoxin for HER2-overexpressing cancer cells was 2.5–3 orders of magnitude lower compared to that for CHO cells not expressing this tumor marker and was 2.5–3 orders of magnitude lower than IC_50_ of free PE40 for HER2-overexpressing cancer cells. These findings provide a basis for expecting in the long run high therapeutic index values of the 4D5scFv-PE40 immunotoxin for its use *in vivo*.

## INTRODUCTION

The progress achieved in studying the molecular basis of carcinogenesis has revealed subtle biochemical differences between tumor and normal cells and, thus, provided opportunities for developing therapies based on these differences. The targeted therapy concept involves the development of drugs specifically interacting with target molecules that are expressed in tumor cells but are not present in normal tissues. This approach enables elimination of tumor cells with minimum negative impact on other tissues and organs.

Targeted agents include, primarily, monoclonal antibodies specifically interacting with surface receptor tumor markers (including anti-angiogenic antibodies) [1, 2] and low-molecular-weight inhibitors of enzymes [3]. An additional toxic component can be introduced into antibody-based drugs to enhance their tumorspecific effect [4]. The drug Kadcyla® [5], which was introduced into clinical practice for the treatment of metastatic breast cancer at the end of 2013, became the first bifunctional agent. This drug is a chemical conjugate of a full-length humanized antibody specific for the HER2 tumor marker and a toxic compound which inhibits association of tubulin subunits during microtubule assembly. In the case where both the targeting and effector (toxic) modules are protein molecules, it becomes possible in principle to combine them into a single polypeptide chain by genetic engineering techniques. Recombinant bifunctional proteins, known as immunotoxins, are exclusively promising molecules for further development of targeted cancer treatment due to their strictly controlled composition, the possibility of biotechnological production in bacterial producers, the possibility of optimization of their properties by genetic engineering techniques, etc. [6].

In this study, we investigated the physico-chemical and functional properties of a new HER2-specific recombinant immunotoxin produced on the basis of a scFv format antibody and *Pseudomonas *exotoxin A.

## MATERIALS AND METHODS

**Preparation and characterization of proteins**

Production of the recombinant immunotoxin 4D5scFv- PE40 and free polypeptides 4D5scFv and PE40 (ETA) was carried out in *E.coli *culture that was preliminarily transformed with the plasmids pSD-4D5scFv-PE40, pSD-4D5scFv, and pSD-PE40 containing genes of the 4D5scFv-PE40, 4D5scFv, and PE40 proteins, respectively, under control of the *lac*-promoter. The plasmids were constructed from the vectors pIG6-4D5 and pIG6-4D5MOCB-ETA [7, 8].

Protein purification was performed in two steps by Ni^2+^-chelate affinity chromatography using a 1 mL HisTrap FF column (GE Healthcare, USA) and ion exchange chromatography on a 1 mL Q Sepharose FF column (GE Healthcare, USA).

Fractions containing the desired protein were analyzed by electrophoresis in 12% PAGE under denaturing conditions according to the standard protocol [9].

The dissociation constant of the complex between 4D5scFv-PE40 and the HER2 receptor was determined by surface plasmon resonance on a BIAcore 3000 optical biosensor (GE Healthcare, USA) using a recombinant extracellular domain of the HER2 receptor p185^HER2-ECD^ (Sino Biological, Inc., China).

**Analysis of cytotoxicity**

In the study, we used the SKOV-3 human ovarian adenocarcinoma cell line (catalog number ATCC-HTB-77), which is characterized by overexpression of the HER2 receptor, and HER2-negative Chinese hamster ovary (CHO) cells (ATCC-CCL-61). To generate the SKOV-kat fluorescent tumor cell line, SKOV-3 cells were transfected with the TurboFP635 red fluorescent protein gene using the pTurboFP635-C vector (Evrogen, Russia) [10].

Cells were grown in a RPMI-1640 medium (HyClone, USA) with 10% fetal calf serum (HyClone, USA) and 2 mM glutamine (PanEco, Russia) at 37 °C under 5% CO_2_.

The cytotoxicity analysis of the studied proteins was performed using the standard MTT assay [11]. In this case, treatment of the cells was conducted in two ways. To evaluate the effect of short-term exposure, the cells were incubated in the presence of the studied proteins for 40 min at 4 °C. Then, the unbound proteins were washed out and the cells were added with the growth medium and grown for 48 h. To analyze the effect of long-term exposure, the cells were grown in the presence of the studied proteins in the medium for 72 h.

The mean value and 95% confidence interval of the protein concentration leading to a 2-fold decrease in the culture viability (IC_50_) were calculated using the GraphPad Prism 6 software.

## RESULTS AND DISCUSSION

**Fig. 1:**
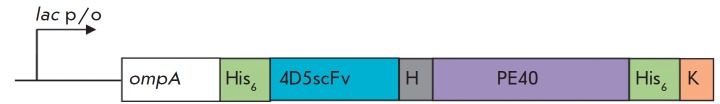
Scheme of recombinant immunotoxin 4D5scFv-PE40. The following encoding regions are shown: ompA (white) is a signal peptide ensuring secretion of the desired recombinant protein to the periplasmic space; His6 (green) is an oligohistidine peptide; 4D5scFv (blue) is the anti-HER2-antibody 4D5scFv; H (gray) is a flexible hydrophilic linker of the hinge region of mouse IgG (16 a.a.); PE40 (lilac) is a fragment of wild-type exotoxin A from *Pseudomonas aeruginosa* (domains II, Ib, and III); K (orange) is the KDEL oligopeptide

The tested immunotoxin 4D5scFv-PE40 is a single polypeptide chain that combines the targeting and toxic modules (*Fig. 1*).

The recombinant protein 4D5scFv-PE40 contains the 40 kDa PE40 fragment of exotoxin A from *Pseudomonas aeruginosa*, lacking the wild-type receptorrecognizing domain. The PE40 fragment is linked to the C-terminus of the anti-HER2-antibody of scFv format (4D5scFv) via a flexible hydrophilic 16-amino acid linker [12]. Because of this linker, the distance between connected fragments of the protein molecule, 4D5scFv and PE40, amounts to 2.5–2.7 nm, which allows the two protein domains to avoid steric hindrance and to retain their functional properties. The 4D5scFv antibody is a recombinant polypeptide composed of fused variable domains of light and heavy chains of the full-length antibody 4D5 specific for the HER2 tumor marker. The antibody 4D5scFv proved to be an effective targeting agent for generating bifunctional cytotoxic proteins [13-18].

At the C-terminus, the immunotoxin molecule contains the oligopeptide KDEL, which is an endoplasmic reticulum translocation signal. Oligohistidine sequences are fused to both ends of the target protein for its purification by metal-chelate affinity chromatography. The signal peptide ompA provides secretion of the desired recombinant protein to the periplasmic space both for reducing its toxic effect on the cell and for increasing the level of a soluble fraction of the desired protein during biotechnological production in bacterial producers [19].

Successive purification of the recombinant protein by metal-chelate and ion exchange chromatography yielded the immunotoxin 4D5scFv-PE40 (Mr=71 kDa) with purity of more than 96%, the stability of which was confirmed by storage at +4 °C for 3 months; this can be regarded as a very good indicator for protein samples. For this period, preservation of homogeneity and high affinity of the immunotoxin 4D5scFv-PE40 for the HER2 receptor extracellular domain (K_d_ ~ 7 nM) was demonstrated. For comparison, K_d_ of the free antibody 4D5scFv determined also by plasmon resonance is 5.2 nM [20].

**Fig. 2:**
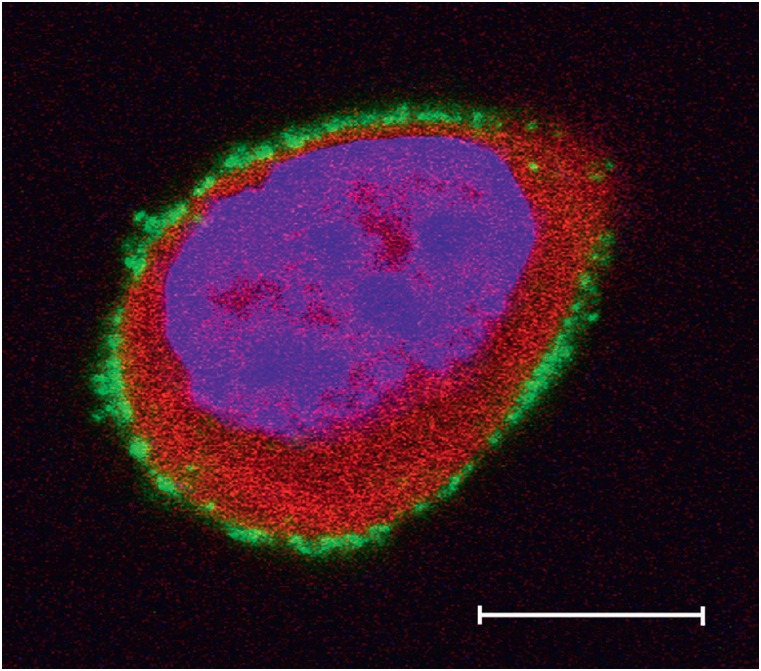
The SKOV-kat cell expressing the red fluorescent protein TurboFP635 (red). The cell nucleus is counterstained with Hoechst 33342 (blue). Expression of the HER2 receptor on the cell surface is confirmed by staining with complexes of quantum dots and the anti-HER2 antibody 4D5scFv [21] (green). The bar is 10 μm

Investigation of the functional properties of 4D5scFv-PE40 was performed in SKOV-kat cell line [10] generated by transfection of the SKOV-3 parent cell line (human ovarian adenocarcinoma), which is characterized by overexpression of the HER2 tumor marker, with the TurboFP635 red fluorescent protein gene. Given the possible impact of transfection on the cells, special attention was paid to preservation of cell morphology and phenotype. Preservation of the HER2 receptor overexpression on the surface of the transfected cells was preliminarily confirmed by immunofluorescence analysis using HER2-targeted semiconductor quantum dots [21] (*Fig. 2*).

Analysis of the 4D5scFv-PE40 cytotoxicity under conditions of short-term (40 min) incubation in the cold demonstrated a highly selective toxic effect on SKOVkat cells (Table). Since these conditions prevent protein internalization, it is obvious that the specific binding to the HER2 receptor and retention on the membrane affect the cell metabolism during subsequent culturing of the cells after immunotoxin removal from the medium.

**Table:** Cytotoxicity of proteins 4D5scFv-PE40, PE40, and 4D5scFv

Cell line	IC_50_, nM*					
	Short-term exposure (40 min)			Long-term exposure (72 h)		
	4D5scFv- PE40	PE40	4D5scFv	4D5scFv- PE40	PE40	4D5scFv
CHO	> 100	> 100	> 100	8.7 (5.6–13.6)	2.9 (1.8–4.6)	> 100
SKOV-kat	22** (5.7–85.3)	> 100	> 100	0.008** (0.006–0.013)	4.9 (1.3–18.4)	> 100
SKOV-3	-	-	-	0.017** (0.011–0.025)	6.6 (3.1–14.0)	> 100

* The mean and 95% confidence interval are presented.

** Statistically significant difference from CHO.

In the body, long-term presence of a drug in the blood and especially in the intercellular matrix is typical.* In vitro *experiments demonstrated that the selectivity of 4D5scFv-PE40 cytotoxic action on SKOV-kat cells persists even for a 72-h incubation of the drug in the growth medium. The 4D5scFv-PE40 concentration causing a twofold decrease in the viability of SKOV-kat cells (IC_50_) is 3 orders of magnitude lower than IC_50_ of 4D5scFv-PE40 for CHO cells not expressing the HER2 receptor. It should be noted that PE40 also exhibits a toxic effect under these conditions; however, IC_50_ of the PE40 polypeptide for SKOV-3 and SKOV-kat cells is also 2.5–3 orders of magnitude higher than IC_50_ of the 4D5scFv-PE40 immunotoxin. These findings provide the basis for expecting in the long run high therapeutic index values of the immunotoxin for its use *in vivo*.

The resistance of SKOV-3, the parent cell line for SKOV-kat, to the action of cytotoxic agents is wellknown [22]. Nevertheless, 4D5scFv-PE40 also exhibited toxicity against SKOV-3 in the picomolar concentration range, which was comparable to that against SKOVkat (Table).

Fluorescent tumor cell lines are unique research tools. Expression of a fluorescent protein by SKOV-kat tumor cells provides an opportunity to continue studying the developed immunotoxin 4D5scFv-PE40 on experimental SKOV-kat-based tumor models using the highly informative methods of intravital whole-body optical imaging.

## CONCLUSIONS

The success of the first targeted anticancer drugs has radically changed the approach to the development of new anticancer agents and led to a change in the standard treatment of many cancers. The biggest success was achieved in oncohematology and the treatment of disseminated tumors; however, the advantage of the targeted approach was also demonstrated in the treatment of solid tumors of a certain molecular profile.

The immunotoxin 4D5scFv-PE40 investigated in this study is designated for targeted therapy of tumors expressing the HER2 tumor marker. The specificity of this immunotoxin is identical to that of the known anticancer drug Herceptin@. At the same time, the presence of a toxic module in 4D5scFv-PE40 multiplies the specific toxicity of the protein against HER2-expressing cells and provides an opportunity to anticipate its effectiveness in further research into the therapeutic potential *in vivo*.

## Abbreviations

HER2: human epidermal growth factor receptor 2
scFv: single-chain variable fragment
PE40: fragment of Pseudomonas exotoxin A
MTT: 3-(4,5-dimethylthiazol-2-yl)-2,5-diphenyltetrazolium bromide
